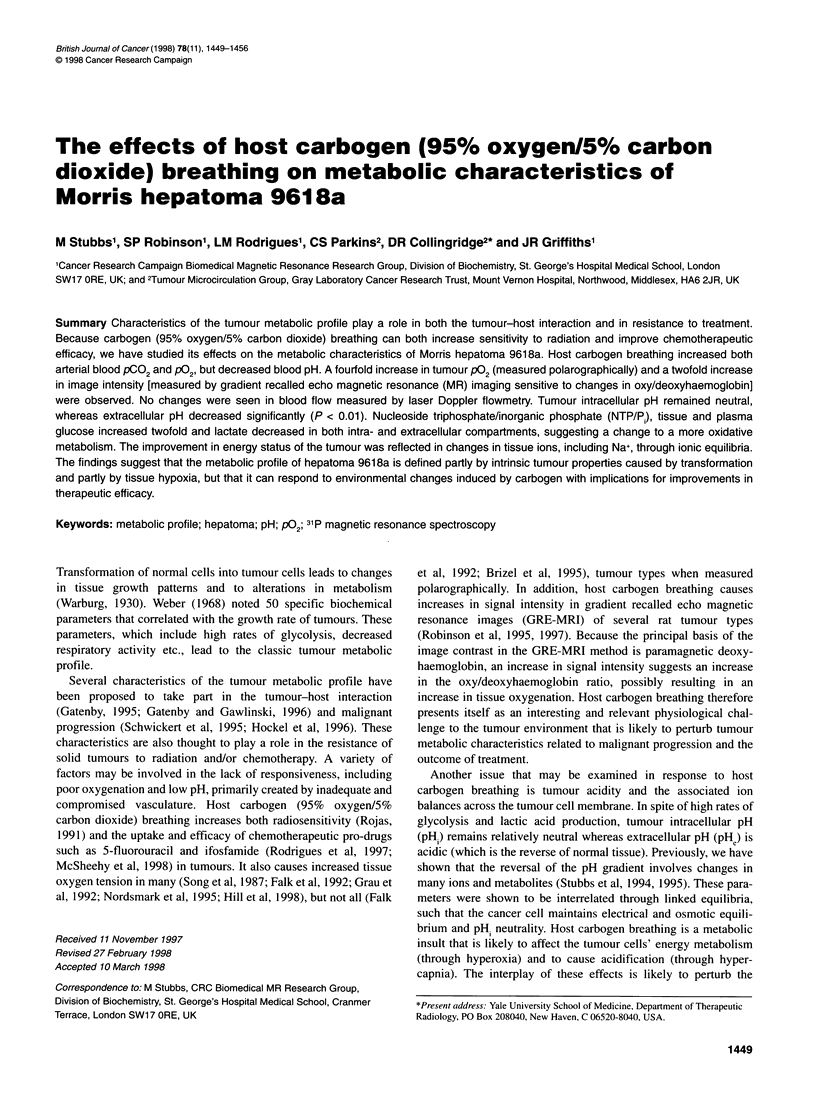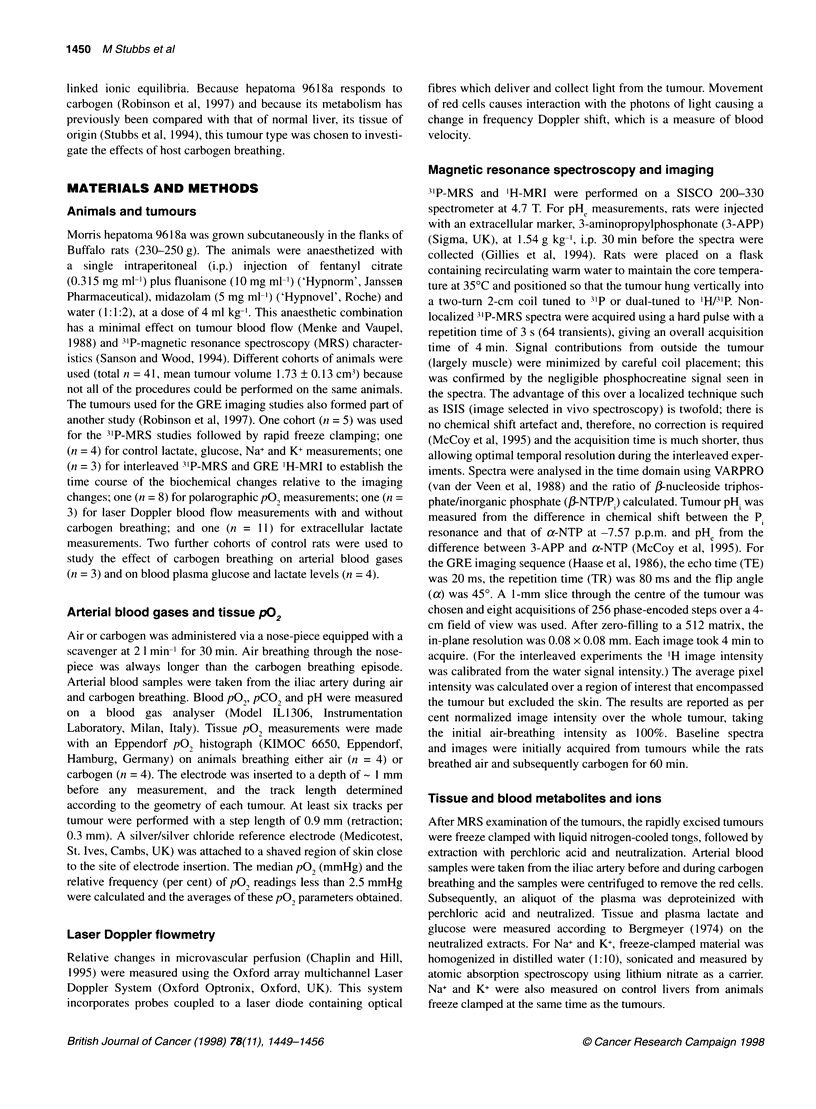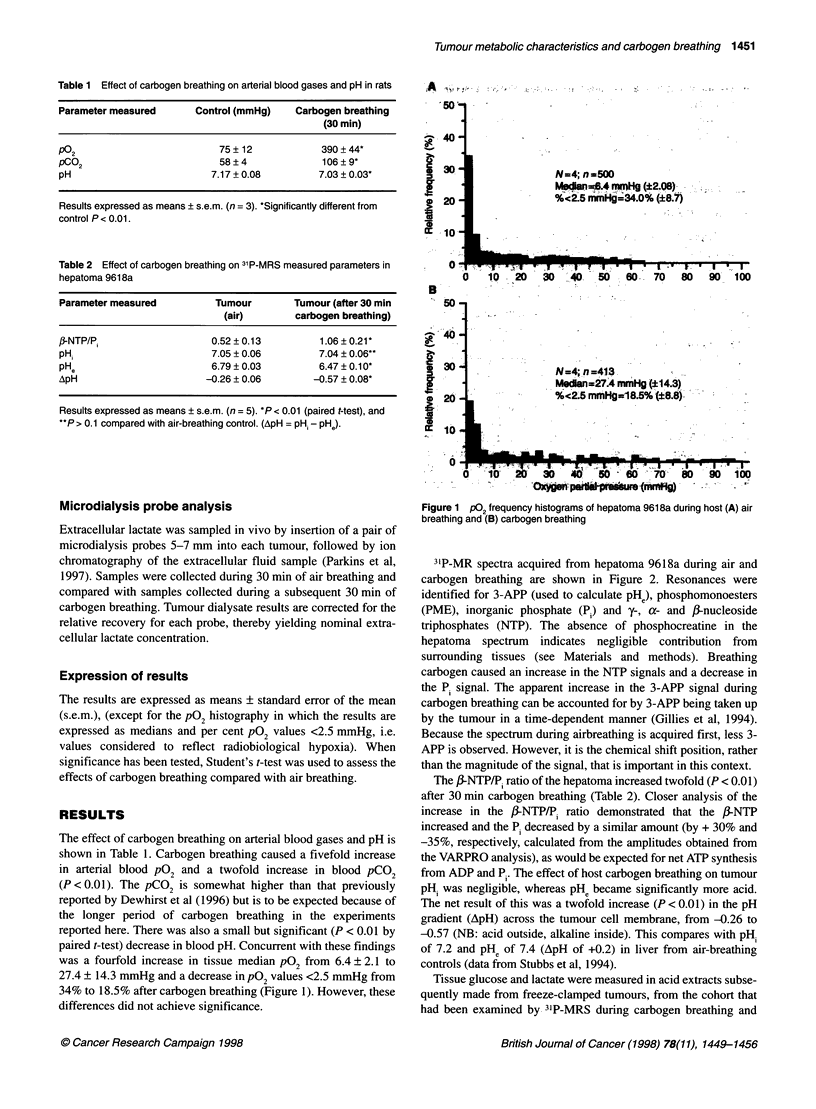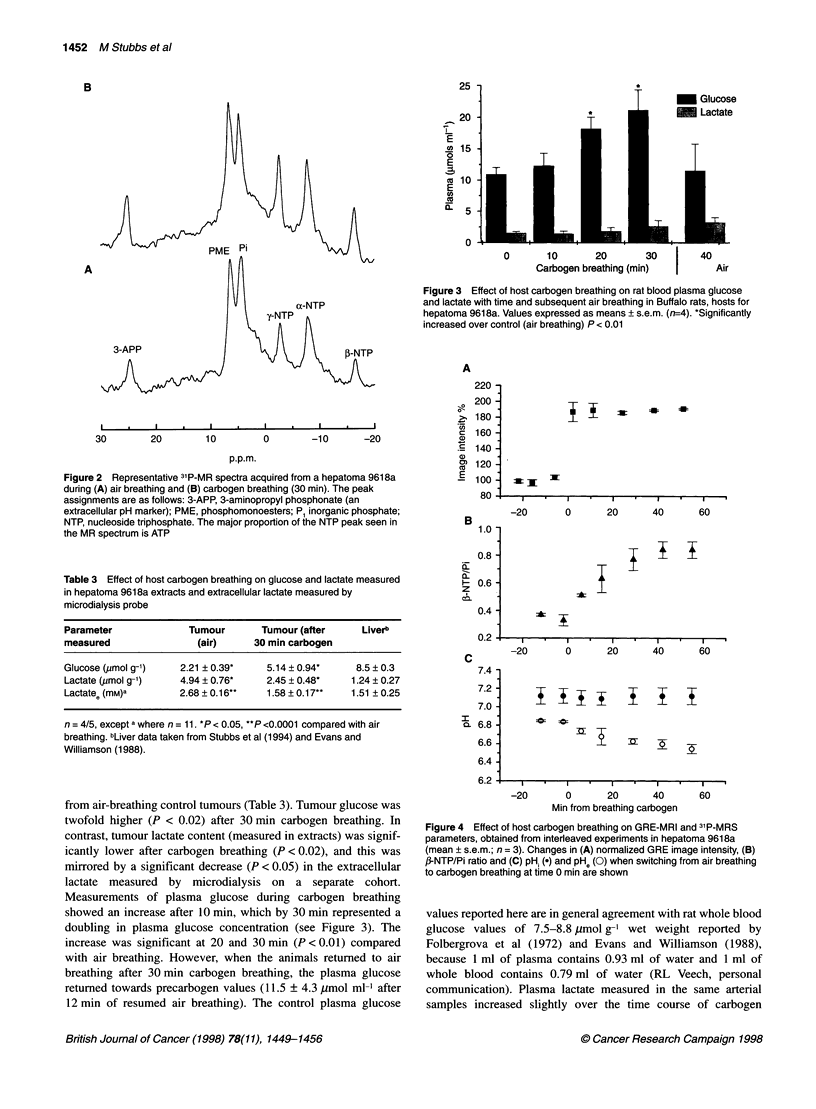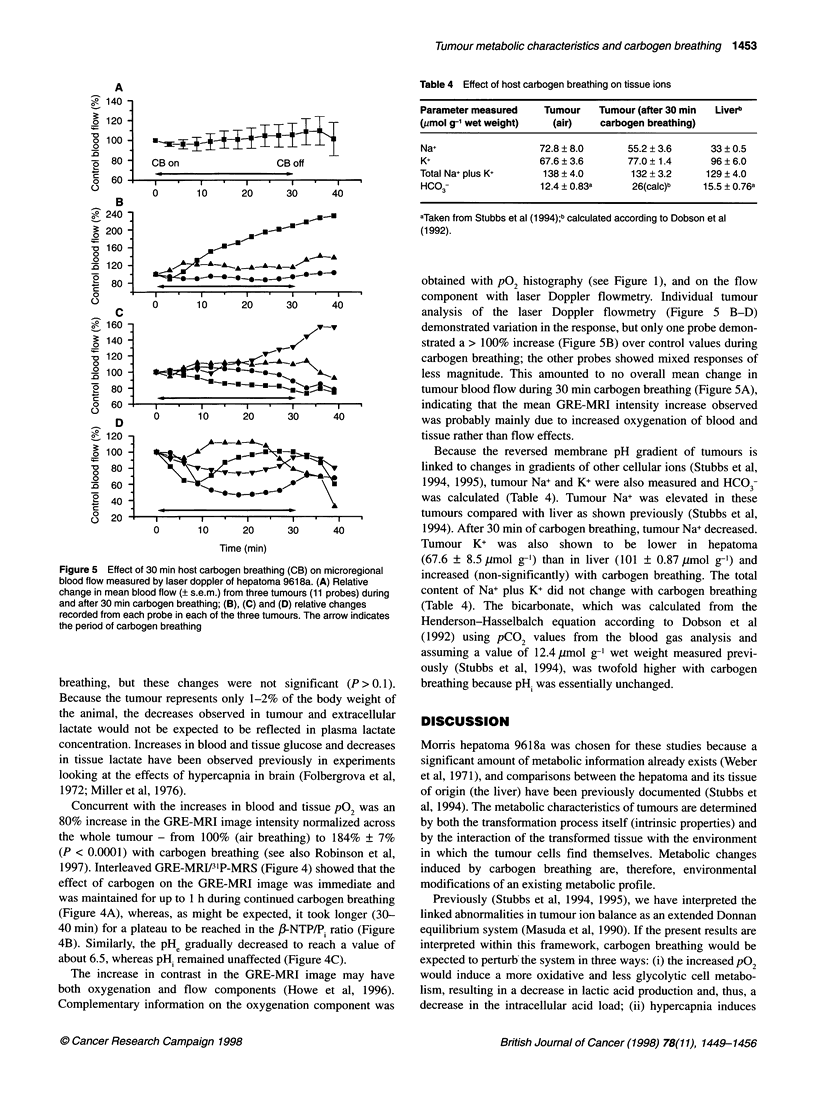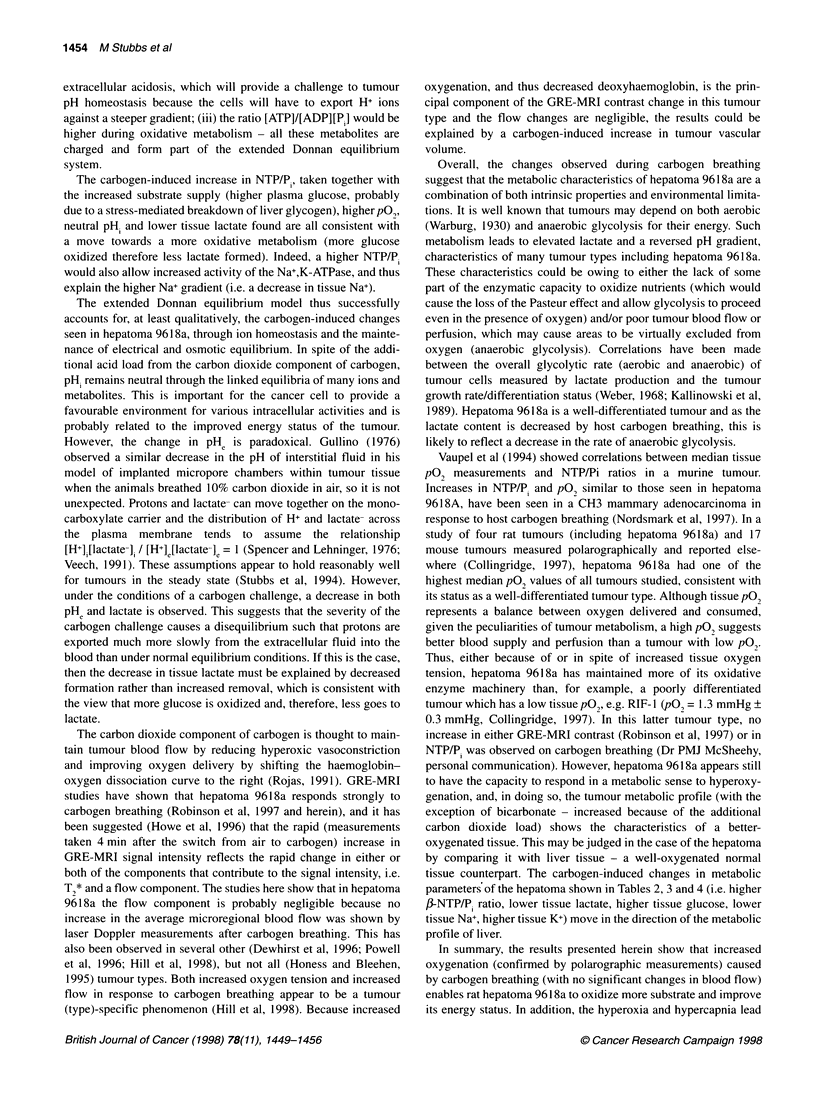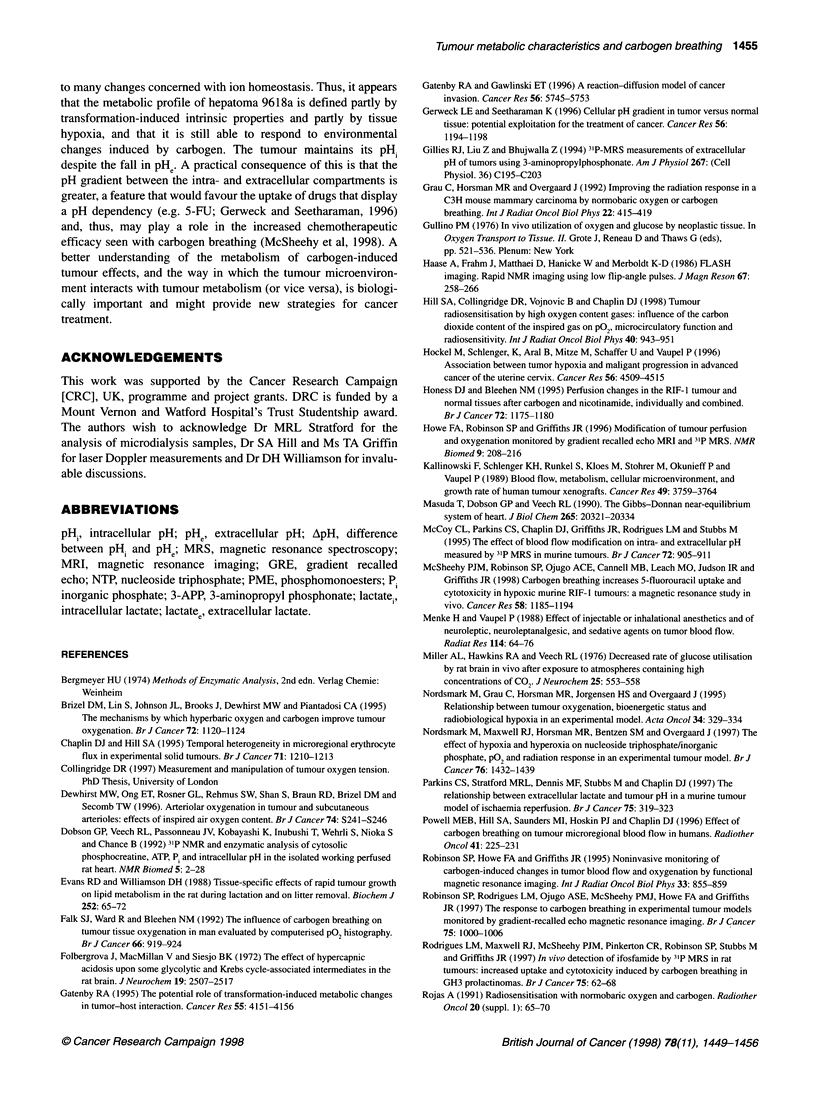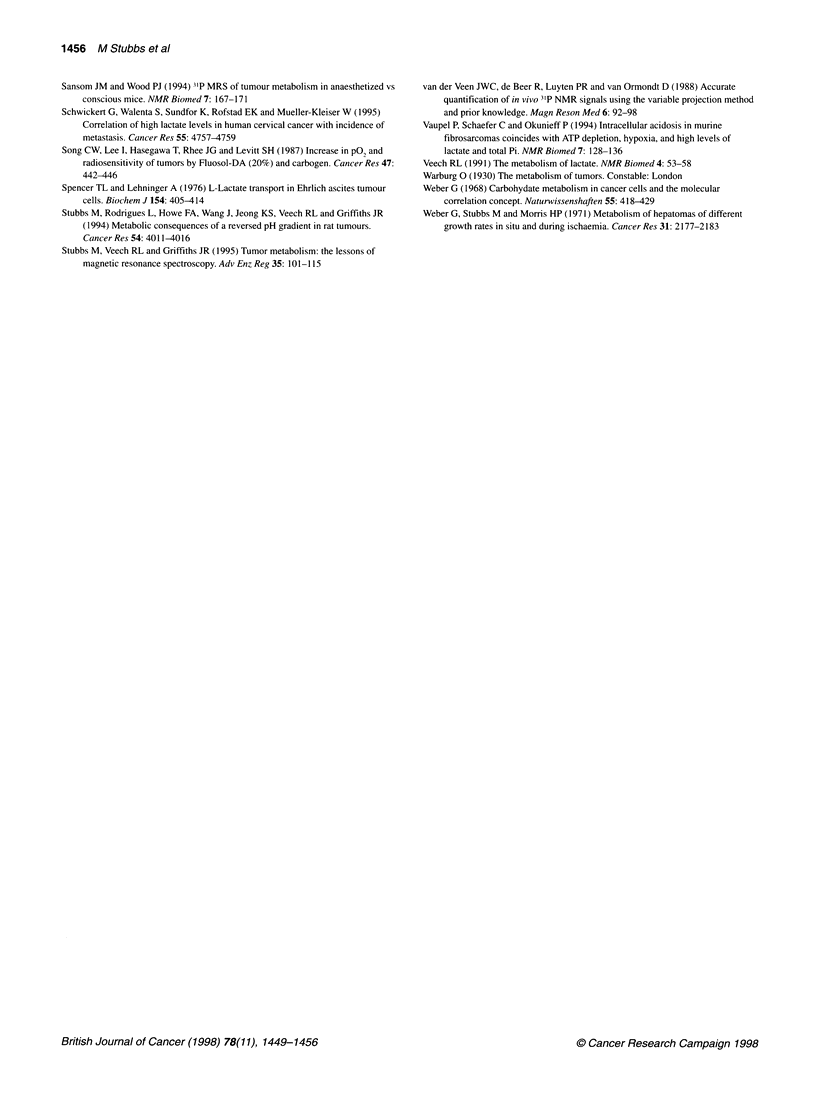# The effects of host carbogen (95% oxygen/5% carbon dioxide) breathing on metabolic characteristics of Morris hepatoma 9618a.

**DOI:** 10.1038/bjc.1998.706

**Published:** 1998-12

**Authors:** M. Stubbs, S. P. Robinson, L. M. Rodrigues, C. S. Parkins, D. R. Collingridge, J. R. Griffiths

**Affiliations:** Cancer Research Campaign Biomedical Magnetic Resonance Research Group, Division of Biochemistry, St. George's Hospital Medical School, London, UK.

## Abstract

Characteristics of the tumour metabolic profile play a role in both the tumour-host interaction and in resistance to treatment. Because carbogen (95% oxygen/5% carbon dioxide) breathing can both increase sensitivity to radiation and improve chemotherapeutic efficacy, we have studied its effects on the metabolic characteristics of Morris hepatoma 9618a. Host carbogen breathing increased both arterial blood pCO2 and pO2, but decreased blood pH. A fourfold increase in tumour pO2 (measured polarographically) and a twofold increase in image intensity [measured by gradient recalled echo magnetic resonance (MR) imaging sensitive to changes in oxy/deoxyhaemoglobin] were observed. No changes were seen in blood flow measured by laser Doppler flowmetry. Tumour intracellular pH remained neutral, whereas extracellular pH decreased significantly (P < 0.01). Nucleoside triphosphate/inorganic phosphate (NTP/Pi), tissue and plasma glucose increased twofold and lactate decreased in both intra- and extracellular compartments, suggesting a change to a more oxidative metabolism. The improvement in energy status of the tumour was reflected in changes in tissue ions, including Na+, through ionic equilibria. The findings suggest that the metabolic profile of hepatoma 9618a is defined partly by intrinsic tumour properties caused by transformation and partly by tissue hypoxia, but that it can respond to environmental changes induced by carbogen with implications for improvements in therapeutic efficacy.


British Journal of Cancer (1998) 78(11), 1449–1456
© 1998 Cancer Research Campaign

### The effects of host carbogen (95% oxygen/5% carbon dioxide) breathing on metabolic characteristics of Morris hepatoma 9618a

**M Stubbs[1], SP Robinson[1], LM Rodrigues[1], CS Parkins[2], DR Collingridge[2]* and JR Griffiths[1]**

[1]Cancer Research Campaign Biomedical Magnetic Resonance Research Group, Division of Biochemistry, St. George's Hospital Medical School, London SW17 0RE, UK; and [2]Tumour Microcirculation Group, Gray Laboratory Cancer Research Trust, Mount Vernon Hospital, Northwood, Middlesex, HA6 2JR, UK

**Summary** Characteristics of the tumour metabolic profile play a role in both the tumour–host interaction and in resistance to treatment. Because carbogen (95% oxygen/5% carbon dioxide) breathing can both increase sensitivity to radiation and improve chemotherapeutic efficacy, we have studied its effects on the metabolic characteristics of Morris hepatoma 9618a. Host carbogen breathing increased both arterial blood $pCO_2$ and $pO_2$, but decreased blood pH. A fourfold increase in tumour $pO_2$ (measured polarographically) and a twofold increase in image intensity [measured by gradient recalled echo magnetic resonance (MR) imaging sensitive to changes in oxy/deoxyhaemoglobin] were observed. No changes were seen in blood flow measured by laser Doppler flowmetry. Tumour intracellular pH remained neutral, whereas extracellular pH decreased significantly ($P < 0.01$). Nucleoside triphosphate/inorganic phosphate (NTP/$P_i$), tissue and plasma glucose increased twofold and lactate decreased in both intra- and extracellular compartments, suggesting a change to a more oxidative metabolism. The improvement in energy status of the tumour was reflected in changes in tissue ions, including $Na^+$, through ionic equilibria. The findings suggest that the metabolic profile of hepatoma 9618a is defined partly by intrinsic tumour properties caused by transformation and partly by tissue hypoxia, but that it can respond to environmental changes induced by carbogen with implications for improvements in therapeutic efficacy.

**Keywords:** metabolic profile; hepatoma; pH; $pO_2$; $^{31}P$ magnetic resonance spectroscopy

Transformation of normal cells into tumour cells leads to changes in tissue growth patterns and to alterations in metabolism (Warburg, 1930). Weber (1968) noted 50 specific biochemical parameters that correlated with the growth rate of tumours. These parameters, which include high rates of glycolysis, decreased respiratory activity etc., lead to the classic tumour metabolic profile.

Several characteristics of the tumour metabolic profile have been proposed to take part in the tumour–host interaction (Gatenby, 1995; Gatenby and Gawlinski, 1996) and malignant progression (Schwickert et al, 1995; Hockel et al, 1996). These characteristics are also thought to play a role in the resistance of solid tumours to radiation and/or chemotherapy. A variety of factors may be involved in the lack of responsiveness, including poor oxygenation and low pH, primarily created by inadequate and compromised vasculature. Host carbogen (95% oxygen/5% carbon dioxide) breathing increases both radiosensitivity (Rojas, 1991) and the uptake and efficacy of chemotherapeutic pro-drugs such as 5-fluorouracil and ifosfamide (Rodrigues et al, 1997; McSheehy et al, 1998) in tumours. It also causes increased tissue oxygen tension in many (Song et al, 1987; Falk et al, 1992; Grau et al, 1992; Nordsmark et al, 1995; Hill et al, 1998), but not all (Falk et al, 1992; Brizel et al, 1995), tumour types when measured polarographically. In addition, host carbogen breathing causes increases in signal intensity in gradient recalled echo magnetic resonance images (GRE-MRI) of several rat tumour types (Robinson et al, 1995, 1997). Because the principal basis of the image contrast in the GRE-MRI method is paramagnetic deoxyhaemoglobin, an increase in signal intensity suggests an increase in the oxy/deoxyhaemoglobin ratio, possibly resulting in an increase in tissue oxygenation. Host carbogen breathing therefore presents itself as an interesting and relevant physiological challenge to the tumour environment that is likely to perturb tumour metabolic characteristics related to malignant progression and the outcome of treatment.

Another issue that may be examined in response to host carbogen breathing is tumour acidity and the associated ion balances across the tumour cell membrane. In spite of high rates of glycolysis and lactic acid production, tumour intracellular pH ($pH_i$) remains relatively neutral whereas extracellular pH ($pH_e$) is acidic (which is the reverse of normal tissue). Previously, we have shown that the reversal of the pH gradient involves changes in many ions and metabolites (Stubbs et al, 1994, 1995). These parameters were shown to be interrelated through linked equilibria, such that the cancer cell maintains electrical and osmotic equilibrium and $pH_i$ neutrality. Host carbogen breathing is a metabolic insult that is likely to affect the tumour cells' energy metabolism (through hyperoxia) and to cause acidification (through hypercapnia). The interplay of these effects is likely to perturb the

*Received 11 November 1997*
*Revised 27 February 1998*
*Accepted 10 March 1998*

*Correspondence to:* M Stubbs, CRC Biomedical MR Research Group, Division of Biochemistry, St. George's Hospital Medical School, Cranmer Terrace, London SW17 0RE, UK

**Present address:* Yale University School of Medicine, Department of Therapeutic Radiology, PO Box 208040, New Haven, C 06520-8040, USA.

linked ionic equilibria. Because hepatoma 9618a responds to carbogen (Robinson et al, 1997) and because its metabolism has previously been compared with that of normal liver, its tissue of origin (Stubbs et al, 1994), this tumour type was chosen to investigate the effects of host carbogen breathing.

### MATERIALS AND METHODS

### Animals and tumours

Morris hepatoma 9618a was grown subcutaneously in the flanks of Buffalo rats (230–250 g). The animals were anaesthetized with a single intraperitoneal (i.p.) injection of fentanyl citrate (0.315 mg $ml^{-1}$) plus fluanisone (10 mg $ml^{-1}$) ('Hypnorm', Janssen Pharmaceutical), midazolam (5 mg $ml^{-1}$) ('Hypnovel', Roche) and water (1:1:2), at a dose of 4 ml $kg^{-1}$. This anaesthetic combination has a minimal effect on tumour blood flow (Menke and Vaupel, 1988) and $^{31}$P-magnetic resonance spectroscopy (MRS) characteristics (Sanson and Wood, 1994). Different cohorts of animals were used (total $n = 41$, mean tumour volume $1.73 \pm 0.13$ $cm^3$) because not all of the procedures could be performed on the same animals. The tumours used for the GRE imaging studies also formed part of another study (Robinson et al, 1997). One cohort ($n = 5$) was used for the $^{31}$P-MRS studies followed by rapid freeze clamping; one ($n = 4$) for control lactate, glucose, $Na^+$ and $K^+$ measurements; one ($n = 3$) for interleaved $^{31}$P-MRS and GRE $^1$H-MRI to establish the time course of the biochemical changes relative to the imaging changes; one ($n = 8$) for polarographic $pO_2$ measurements; one ($n = 3$) for laser Doppler blood flow measurements with and without carbogen breathing; and one ($n = 11$) for extracellular lactate measurements. Two further cohorts of control rats were used to study the effect of carbogen breathing on arterial blood gases ($n = 3$) and on blood plasma glucose and lactate levels ($n = 4$).

### Arterial blood gases and tissue $pO_2$

Air or carbogen was administered via a nose-piece equipped with a scavenger at 2 l $min^{-1}$ for 30 min. Air breathing through the nose-piece was always longer than the carbogen breathing episode. Arterial blood samples were taken from the iliac artery during air and carbogen breathing. Blood $pO_2$, $pCO_2$ and pH were measured on a blood gas analyser (Model IL1306, Instrumentation Laboratory, Milan, Italy). Tissue $pO_2$ measurements were made with an Eppendorf $pO_2$ histograph (KIMOC 6650, Eppendorf, Hamburg, Germany) on animals breathing either air ($n = 4$) or carbogen ($n = 4$). The electrode was inserted to a depth of ~ 1 mm before any measurement, and the track length determined according to the geometry of each tumour. At least six tracks per tumour were performed with a step length of 0.9 mm (retraction; 0.3 mm). A silver/silver chloride reference electrode (Medicotest, St. Ives, Cambs, UK) was attached to a shaved region of skin close to the site of electrode insertion. The median $pO_2$ (mmHg) and the relative frequency (per cent) of $pO_2$ readings less than 2.5 mmHg were calculated and the averages of these $pO_2$ parameters obtained.

### Laser Doppler flowmetry

Relative changes in microvascular perfusion (Chaplin and Hill, 1995) were measured using the Oxford array multichannel Laser Doppler System (Oxford Optronix, Oxford, UK). This system incorporates probes coupled to a laser diode containing optical fibres which deliver and collect light from the tumour. Movement of red cells causes interaction with the photons of light causing a change in frequency Doppler shift, which is a measure of blood velocity.

### Magnetic resonance spectroscopy and imaging

$^{31}$P-MRS and $^1$H-MRI were performed on a SISCO 200–330 spectrometer at 4.7 T. For $pH_e$ measurements, rats were injected with an extracellular marker, 3-aminopropylphosphonate (3-APP) (Sigma, UK), at 1.54 g $kg^{-1}$, i.p. 30 min before the spectra were collected (Gillies et al, 1994). Rats were placed on a flask containing recirculating warm water to maintain the core temperature at 35°C and positioned so that the tumour hung vertically into a two-turn 2-cm coil tuned to $^{31}$P or dual-tuned to $^1$H/$^{31}$P. Non-localized $^{31}$P-MRS spectra were acquired using a hard pulse with a repetition time of 3 s (64 transients), giving an overall acquisition time of 4 min. Signal contributions from outside the tumour (largely muscle) were minimized by careful coil placement; this was confirmed by the negligible phosphocreatine signal seen in the spectra. The advantage of this over a localized technique such as ISIS (image selected in vivo spectroscopy) is twofold; there is no chemical shift artefact and, therefore, no correction is required (McCoy et al, 1995) and the acquisition time is much shorter, thus allowing optimal temporal resolution during the interleaved experiments. Spectra were analysed in the time domain using VARPRO (van der Veen et al, 1988) and the ratio of $\beta$-nucleoside triphosphate/inorganic phosphate ($\beta$-NTP/$P_i$) calculated. Tumour $pH_i$ was measured from the difference in chemical shift between the $P_i$ resonance and that of $\alpha$-NTP at –7.57 p.p.m. and $pH_e$ from the difference between 3-APP and $\alpha$-NTP (McCoy et al, 1995). For the GRE imaging sequence (Haase et al, 1986), the echo time (TE) was 20 ms, the repetition time (TR) was 80 ms and the flip angle ($\alpha$) was 45°. A 1-mm slice through the centre of the tumour was chosen and eight acquisitions of 256 phase-encoded steps over a 4-cm field of view was used. After zero-filling to a 512 matrix, the in-plane resolution was $0.08 \times 0.08$ mm. Each image took 4 min to acquire. (For the interleaved experiments the $^1$H image intensity was calibrated from the water signal intensity.) The average pixel intensity was calculated over a region of interest that encompassed the tumour but excluded the skin. The results are reported as per cent normalized image intensity over the whole tumour, taking the initial air-breathing intensity as 100%. Baseline spectra and images were initially acquired from tumours while the rats breathed air and subsequently carbogen for 60 min.

### Tissue and blood metabolites and ions

After MRS examination of the tumours, the rapidly excised tumours were freeze clamped with liquid nitrogen-cooled tongs, followed by extraction with perchloric acid and neutralization. Arterial blood samples were taken from the iliac artery before and during carbogen breathing and the samples were centrifuged to remove the red cells. Subsequently, an aliquot of the plasma was deproteinized with perchloric acid and neutralized. Tissue and plasma lactate and glucose were measured according to Bergmeyer (1974) on the neutralized extracts. For $Na^+$ and $K^+$, freeze-clamped material was homogenized in distilled water (1:10), sonicated and measured by atomic absorption spectroscopy using lithium nitrate as a carrier. $Na^+$ and $K^+$ were also measured on control livers from animals freeze clamped at the same time as the tumours.

© Cancer Research Campaign 1998

**Table 1** Effect of carbogen breathing on arterial blood gases and pH in rats

| Parameter measured | Control (mmHg) | Carbogen breathing (30 min) |
|---|---|---|
| $pO_2$ | 75 ± 12 | 390 ± 44* |
| $pCO_2$ | 58 ± 4 | 106 ± 9* |
| pH | 7.17 ± 0.08 | 7.03 ± 0.03* |

Results expressed as means ± s.e.m. ($n$ = 3). *Significantly different from control $P < 0.01$.

**Table 2** Effect of carbogen breathing on $^{31}$P-MRS measured parameters in hepatoma 9618a

| Parameter measured | Tumour (air) | Tumour (after 30 min carbogen breathing) |
|---|---|---|
| $\beta$-NTP/$P_i$ | 0.52 ± 0.13 | 1.06 ± 0.21* |
| $pH_i$ | 7.05 ± 0.06 | 7.04 ± 0.06** |
| $pH_e$ | 6.79 ± 0.03 | 6.47 ± 0.10* |
| ΔpH | –0.26 ± 0.06 | –0.57 ± 0.08* |

Results expressed as means ± s.e.m. ($n$ = 5). *$P < 0.01$ (paired $t$-test), and **$P > 0.1$ compared with air-breathing control. (ΔpH = $pH_i - pH_e$).

**Figure 1** $pO_2$ frequency histograms of hepatoma 9618a during host (**A**) air breathing and (**B**) carbogen breathing

### Microdialysis probe analysis

Extracellular lactate was sampled in vivo by insertion of a pair of microdialysis probes 5–7 mm into each tumour, followed by ion chromatography of the extracellular fluid sample (Parkins et al, 1997). Samples were collected during 30 min of air breathing and compared with samples collected during a subsequent 30 min of carbogen breathing. Tumour dialysate results are corrected for the relative recovery for each probe, thereby yielding nominal extracellular lactate concentration.

### Expression of results

The results are expressed as means ± standard error of the mean (s.e.m.), (except for the $pO_2$ histography in which the results are expressed as medians and per cent $pO_2$ values <2.5 mmHg, i.e. values considered to reflect radiobiological hypoxia). When significance has been tested, Student's $t$-test was used to assess the effects of carbogen breathing compared with air breathing.

### RESULTS

The effect of carbogen breathing on arterial blood gases and pH is shown in Table 1. Carbogen breathing caused a fivefold increase in arterial blood $pO_2$ and a twofold increase in blood $pCO_2$ ($P < 0.01$). The $pCO_2$ is somewhat higher than that previously reported by Dewhirst et al (1996) but is to be expected because of the longer period of carbogen breathing in the experiments reported here. There was also a small but significant ($P < 0.01$ by paired $t$-test) decrease in blood pH. Concurrent with these findings was a fourfold increase in tissue median $pO_2$ from 6.4 ± 2.1 to 27.4 ± 14.3 mmHg and a decrease in $pO_2$ values <2.5 mmHg from 34% to 18.5% after carbogen breathing (Figure 1). However, these differences did not achieve significance.

$^{31}$P-MR spectra acquired from hepatoma 9618a during air and carbogen breathing are shown in Figure 2. Resonances were identified for 3-APP (used to calculate $pH_e$), phosphomonoesters (PME), inorganic phosphate ($P_i$) and $\gamma$-, $\alpha$- and $\beta$-nucleoside triphosphates (NTP). The absence of phosphocreatine in the hepatoma spectrum indicates negligible contribution from surrounding tissues (see Materials and methods). Breathing carbogen caused an increase in the NTP signals and a decrease in the $P_i$ signal. The apparent increase in the 3-APP signal during carbogen breathing can be accounted for by 3-APP being taken up by the tumour in a time-dependent manner (Gillies et al, 1994). Because the spectrum during airbreathing is acquired first, less 3-APP is observed. However, it is the chemical shift position, rather than the magnitude of the signal, that is important in this context.

The $\beta$-NTP/$P_i$ ratio of the hepatoma increased twofold ($P < 0.01$) after 30 min carbogen breathing (Table 2). Closer analysis of the increase in the $\beta$-NTP/$P_i$ ratio demonstrated that the $\beta$-NTP increased and the $P_i$ decreased by a similar amount (by + 30% and –35%, respectively, calculated from the amplitudes obtained from the VARPRO analysis), as would be expected for net ATP synthesis from ADP and $P_i$. The effect of host carbogen breathing on tumour $pH_i$ was negligible, whereas $pH_e$ became significantly more acid. The net result of this was a twofold increase ($P < 0.01$) in the pH gradient (ΔpH) across the tumour cell membrane, from –0.26 to –0.57 (NB: acid outside, alkaline inside). This compares with $pH_i$ of 7.2 and $pH_e$ of 7.4 (ΔpH of +0.2) in liver from air-breathing controls (data from Stubbs et al, 1994).

Tissue glucose and lactate were measured in acid extracts subsequently made from freeze-clamped tumours, from the cohort that had been examined by $^{31}$P-MRS during carbogen breathing and

© Cancer Research Campaign 1998

Figure 2 Representative $^{31}$P-MR spectra acquired from a hepatoma 9618a during (A) air breathing and (B) carbogen breathing (30 min). The peak assignments are as follows: 3-APP, 3-aminopropyl phosphonate (an extracellular pH marker); PME, phosphomonoesters; $P_i$ inorganic phosphate; NTP, nucleoside triphosphate. The major proportion of the NTP peak seen in the MR spectrum is ATP

Table 3 Effect of host carbogen breathing on glucose and lactate measured in hepatoma 9618a extracts and extracellular lactate measured by microdialysis probe

| Parameter measured | Tumour (air) | Tumour (after 30 min carbogen | Liver[b] |
|---|---|---|---|
| Glucose (μmol g$^{-1}$) | 2.21 ± 0.39* | 5.14 ± 0.94* | 8.5 ± 0.3 |
| Lactate (μmol g$^{-1}$) | 4.94 ± 0.76* | 2.45 ± 0.48* | 1.24 ± 0.27 |
| Lactate$_e$ (mM)[a] | 2.68 ± 0.16** | 1.58 ± 0.17** | 1.51 ± 0.25 |

$n$ = 4/5, except [a] where $n$ = 11. *$P$ < 0.05, **$P$ <0.0001 compared with air breathing. [b]Liver data taken from Stubbs et al (1994) and Evans and Williamson (1988).

Figure 3 Effect of host carbogen breathing on rat blood plasma glucose and lactate with time and subsequent air breathing in Buffalo rats, hosts for hepatoma 9618a. Values expressed as means ± s.e.m. ($n$=4). *Significantly increased over control (air breathing) $P$ < 0.01

Figure 4 Effect of host carbogen breathing on GRE-MRI and $^{31}$P-MRS parameters, obtained from interleaved experiments in hepatoma 9618a (mean ± s.e.m.; $n$ = 3). Changes in (A) normalized GRE image intensity, (B) β-NTP/Pi ratio and (C) $pH_i$ (•) and $pH_e$ (○) when switching from air breathing to carbogen breathing at time 0 min are shown from air-breathing control tumours (Table 3). Tumour glucose was twofold higher ($P$ < 0.02) after 30 min carbogen breathing. In contrast, tumour lactate content (measured in extracts) was significantly lower after carbogen breathing ($P$ < 0.02), and this was mirrored by a significant decrease ($P$ < 0.05) in the extracellular lactate measured by microdialysis on a separate cohort. Measurements of plasma glucose during carbogen breathing showed an increase after 10 min, which by 30 min represented a doubling in plasma glucose concentration (see Figure 3). The increase was significant at 20 and 30 min ($P$ < 0.01) compared with air breathing. However, when the animals returned to air breathing after 30 min carbogen breathing, the plasma glucose returned towards precarbogen values (11.5 ± 4.3 μmol ml$^{-1}$ after 12 min of resumed air breathing). The control plasma glucose values reported here are in general agreement with rat whole blood glucose values of 7.5–8.8 μmol g$^{-1}$ wet weight reported by Folbergrova et al (1972) and Evans and Williamson (1988), because 1 ml of plasma contains 0.93 ml of water and 1 ml of whole blood contains 0.79 ml of water (RL Veech, personal communication). Plasma lactate measured in the same arterial samples increased slightly over the time course of carbogen

© Cancer Research Campaign 1998

**Figure 5** Effect of 30 min host carbogen breathing (CB) on microregional blood flow measured by laser doppler of hepatoma 9618a. (**A**) Relative change in mean blood flow (± s.e.m.) from three tumours (11 probes) during and after 30 min carbogen breathing; (**B**), (**C**) and (**D**) relative changes recorded from each probe in each of the three tumours. The arrow indicates the period of carbogen breathing breathing, but these changes were not significant ($P > 0.1$). Because the tumour represents only 1–2% of the body weight of the animal, the decreases observed in tumour and extracellular lactate would not be expected to be reflected in plasma lactate concentration. Increases in blood and tissue glucose and decreases in tissue lactate have been observed previously in experiments looking at the effects of hypercapnia in brain (Folbergrova et al, 1972; Miller et al, 1976).

Concurrent with the increases in blood and tissue $pO_2$ was an 80% increase in the GRE-MRI image intensity normalized across the whole tumour – from 100% (air breathing) to 184% ± 7% ($P < 0.0001$) with carbogen breathing (see also Robinson et al, 1997). Interleaved GRE-MRI/$^{31}$P-MRS (Figure 4) showed that the effect of carbogen on the GRE-MRI image was immediate and was maintained for up to 1 h during continued carbogen breathing (Figure 4A), whereas, as might be expected, it took longer (30–40 min) for a plateau to be reached in the $\beta$-NTP/P$_i$ ratio (Figure 4B). Similarly, the pH$_e$ gradually decreased to reach a value of about 6.5, whereas pH$_i$ remained unaffected (Figure 4C).

The increase in contrast in the GRE-MRI image may have both oxygenation and flow components (Howe et al, 1996). Complementary information on the oxygenation component was obtained with $pO_2$ histography (see Figure 1), and on the flow component with laser Doppler flowmetry. Individual tumour analysis of the laser Doppler flowmetry (Figure 5 B–D) demonstrated variation in the response, but only one probe demonstrated a > 100% increase (Figure 5B) over control values during carbogen breathing; the other probes showed mixed responses of less magnitude. This amounted to no overall mean change in tumour blood flow during 30 min carbogen breathing (Figure 5A), indicating that the mean GRE-MRI intensity increase observed was probably mainly due to increased oxygenation of blood and tissue rather than flow effects.

**Table 4** Effect of host carbogen breathing on tissue ions

| Parameter measured ($\mu$mol g$^{-1}$ wet weight) | Tumour (air) | Tumour (after 30 min carbogen breathing) | Liver[b] |
|---|---|---|---|
| $Na^+$ | 72.8 ± 8.0 | 55.2 ± 3.6 | 33 ± 0.5 |
| $K^+$ | 67.6 ± 3.6 | 77.0 ± 1.4 | 96 ± 6.0 |
| Total $Na^+$ plus $K^+$ | 138 ± 4.0 | 132 ± 3.2 | 129 ± 4.0 |
| $HCO_3^-$ | 12.4 ± 0.83[a] | 26(calc)[b] | 15.5 ± 0.76[a] |

[a]Taken from Stubbs et al (1994);[b] calculated according to Dobson et al (1992).

Because the reversed membrane pH gradient of tumours is linked to changes in gradients of other cellular ions (Stubbs et al, 1994, 1995), tumour $Na^+$ and $K^+$ were also measured and $HCO_3^-$ was calculated (Table 4). Tumour $Na^+$ was elevated in these tumours compared with liver as shown previously (Stubbs et al, 1994). After 30 min of carbogen breathing, tumour $Na^+$ decreased. Tumour $K^+$ was also shown to be lower in hepatoma (67.6 ± 8.5 $\mu$mol g$^{-1}$) than in liver (101 ± 0.87 $\mu$mol g$^{-1}$) and increased (non-significantly) with carbogen breathing. The total content of $Na^+$ plus $K^+$ did not change with carbogen breathing (Table 4). The bicarbonate, which was calculated from the Henderson–Hasselbalch equation according to Dobson et al (1992) using $pCO_2$ values from the blood gas analysis and assuming a value of 12.4 $\mu$mol g$^{-1}$ wet weight measured previously (Stubbs et al, 1994), was twofold higher with carbogen breathing because pH$_i$ was essentially unchanged.

### DISCUSSION

Morris hepatoma 9618a was chosen for these studies because a significant amount of metabolic information already exists (Weber et al, 1971), and comparisons between the hepatoma and its tissue of origin (the liver) have been previously documented (Stubbs et al, 1994). The metabolic characteristics of tumours are determined by both the transformation process itself (intrinsic properties) and by the interaction of the transformed tissue with the environment in which the tumour cells find themselves. Metabolic changes induced by carbogen breathing are, therefore, environmental modifications of an existing metabolic profile.

Previously (Stubbs et al, 1994, 1995), we have interpreted the linked abnormalities in tumour ion balance as an extended Donnan equilibrium system (Masuda et al, 1990). If the present results are interpreted within this framework, carbogen breathing would be expected to perturb the system in three ways: (i) the increased $pO_2$ would induce a more oxidative and less glycolytic cell metabolism, resulting in a decrease in lactic acid production and, thus, a decrease in the intracellular acid load; (ii) hypercapnia induces

© Cancer Research Campaign 1998

extracellular acidosis, which will provide a challenge to tumour pH homeostasis because the cells will have to export $H^+$ ions against a steeper gradient; (iii) the ratio [ATP]/[ADP][$P_i$] would be higher during oxidative metabolism – all these metabolites are charged and form part of the extended Donnan equilibrium system.

The carbogen-induced increase in NTP/$P_i$, taken together with the increased substrate supply (higher plasma glucose, probably due to a stress-mediated breakdown of liver glycogen), higher $pO_2$, neutral $pH_i$ and lower tissue lactate found are all consistent with a move towards a more oxidative metabolism (more glucose oxidized therefore less lactate formed). Indeed, a higher NTP/$P_i$ would also allow increased activity of the $Na^+$,$K$-ATPase, and thus explain the higher $Na^+$ gradient (i.e. a decrease in tissue $Na^+$).

The extended Donnan equilibrium model thus successfully accounts for, at least qualitatively, the carbogen-induced changes seen in hepatoma 9618a, through ion homeostasis and the maintenance of electrical and osmotic equilibrium. In spite of the additional acid load from the carbon dioxide component of carbogen, $pH_i$ remains neutral through the linked equilibria of many ions and metabolites. This is important for the cancer cell to provide a favourable environment for various intracellular activities and is probably related to the improved energy status of the tumour. However, the change in $pH_e$ is paradoxical. Gullino (1976) observed a similar decrease in the pH of interstitial fluid in his model of implanted micropore chambers within tumour tissue when the animals breathed 10% carbon dioxide in air, so it is not unexpected. Protons and lactate$^-$ can move together on the monocarboxylate carrier and the distribution of $H^+$ and lactate$^-$ across the plasma membrane tends to assume the relationship $[H^+]_i[\text{lactate}^-]_i$ / $[H^+]_e[\text{lactate}^-]_e = 1$ (Spencer and Lehninger, 1976; Veech, 1991). These assumptions appear to hold reasonably well for tumours in the steady state (Stubbs et al, 1994). However, under the conditions of a carbogen challenge, a decrease in both $pH_e$ and lactate is observed. This suggests that the severity of the carbogen challenge causes a disequilibrium such that protons are exported much more slowly from the extracellular fluid into the blood than under normal equilibrium conditions. If this is the case, then the decrease in tissue lactate must be explained by decreased formation rather than increased removal, which is consistent with the view that more glucose is oxidized and, therefore, less goes to lactate.

The carbon dioxide component of carbogen is thought to maintain tumour blood flow by reducing hyperoxic vasoconstriction and improving oxygen delivery by shifting the haemoglobin–oxygen dissociation curve to the right (Rojas, 1991). GRE-MRI studies have shown that hepatoma 9618a responds strongly to carbogen breathing (Robinson et al, 1997 and herein), and it has been suggested (Howe et al, 1996) that the rapid (measurements taken 4 min after the switch from air to carbogen) increase in GRE-MRI signal intensity reflects the rapid change in either or both of the components that contribute to the signal intensity, i.e. $T_2$* and a flow component. The studies here show that in hepatoma 9618a the flow component is probably negligible because no increase in the average microregional blood flow was shown by laser Doppler measurements after carbogen breathing. This has also been observed in several other (Dewhirst et al, 1996; Powell et al, 1996; Hill et al, 1998), but not all (Honess and Bleehen, 1995) tumour types. Both increased oxygen tension and increased flow in response to carbogen breathing appear to be a tumour (type)-specific phenomenon (Hill et al, 1998). Because increased oxygenation, and thus decreased deoxyhaemoglobin, is the principal component of the GRE-MRI contrast change in this tumour type and the flow changes are negligible, the results could be explained by a carbogen-induced increase in tumour vascular volume.

Overall, the changes observed during carbogen breathing suggest that the metabolic characteristics of hepatoma 9618a are a combination of both intrinsic properties and environmental limitations. It is well known that tumours may depend on both aerobic (Warburg, 1930) and anaerobic glycolysis for their energy. Such metabolism leads to elevated lactate and a reversed pH gradient, characteristics of many tumour types including hepatoma 9618a. These characteristics could be owing to either the lack of some part of the enzymatic capacity to oxidize nutrients (which would cause the loss of the Pasteur effect and allow glycolysis to proceed even in the presence of oxygen) and/or poor tumour blood flow or perfusion, which may cause areas to be virtually excluded from oxygen (anaerobic glycolysis). Correlations have been made between the overall glycolytic rate (aerobic and anaerobic) of tumour cells measured by lactate production and the tumour growth rate/differentiation status (Weber, 1968; Kallinowski et al, 1989). Hepatoma 9618a is a well-differentiated tumour and as the lactate content is decreased by host carbogen breathing, this is likely to reflect a decrease in the rate of anaerobic glycolysis.

Vaupel et al (1994) showed correlations between median tissue $pO_2$ measurements and NTP/Pi ratios in a murine tumour. Increases in NTP/$P_i$ and $pO_2$ similar to those seen in hepatoma 9618A, have been seen in a CH3 mammary adenocarcinoma in response to host carbogen breathing (Nordsmark et al, 1997). In a study of four rat tumours (including hepatoma 9618a) and 17 mouse tumours measured polarographically and reported elsewhere (Collingridge, 1997), hepatoma 9618a had one of the highest median $pO_2$ values of all tumours studied, consistent with its status as a well-differentiated tumour type. Although tissue $pO_2$ represents a balance between oxygen delivered and consumed, given the peculiarities of tumour metabolism, a high $pO_2$ suggests better blood supply and perfusion than a tumour with low $pO_2$. Thus, either because of or in spite of increased tissue oxygen tension, hepatoma 9618a has maintained more of its oxidative enzyme machinery than, for example, a poorly differentiated tumour which has a low tissue $pO_2$, e.g. RIF-1 ($pO_2$ = 1.3 mmHg ± 0.3 mmHg, Collingridge, 1997). In this latter tumour type, no increase in either GRE-MRI contrast (Robinson et al, 1997) or in NTP/$P_i$ was observed on carbogen breathing (Dr PMJ McSheehy, personal communication). However, hepatoma 9618a appears still to have the capacity to respond in a metabolic sense to hyperoxygenation, and, in doing so, the tumour metabolic profile (with the exception of bicarbonate – increased because of the additional carbon dioxide load) shows the characteristics of a better-oxygenated tissue. This may be judged in the case of the hepatoma by comparing it with liver tissue – a well-oxygenated normal tissue counterpart. The carbogen-induced changes in metabolic parameters of the hepatoma shown in Tables 2, 3 and 4 (i.e. higher $\beta$-NTP/$P_i$ ratio, lower tissue lactate, higher tissue glucose, lower tissue $Na^+$, higher tissue $K^+$) move in the direction of the metabolic profile of liver.

In summary, the results presented herein show that increased oxygenation (confirmed by polarographic measurements) caused by carbogen breathing (with no significant changes in blood flow) enables rat hepatoma 9618a to oxidize more substrate and improve its energy status. In addition, the hyperoxia and hypercapnia lead

*© Cancer Research Campaign 1998*

to many changes concerned with ion homeostasis. Thus, it appears that the metabolic profile of hepatoma 9618a is defined partly by transformation-induced intrinsic properties and partly by tissue hypoxia, and that it is still able to respond to environmental changes induced by carbogen. The tumour maintains its $pH_i$ despite the fall in $pH_e$. A practical consequence of this is that the pH gradient between the intra- and extracellular compartments is greater, a feature that would favour the uptake of drugs that display a pH dependency (e.g. 5-FU; Gerweck and Seetharaman, 1996) and, thus, may play a role in the increased chemotherapeutic efficacy seen with carbogen breathing (McSheehy et al, 1998). A better understanding of the metabolism of carbogen-induced tumour effects, and the way in which the tumour microenvironment interacts with tumour metabolism (or vice versa), is biologically important and might provide new strategies for cancer treatment.

### ACKNOWLEDGEMENTS

This work was supported by the Cancer Research Campaign [CRC], UK, programme and project grants. DRC is funded by a Mount Vernon and Watford Hospital's Trust Studentship award. The authors wish to acknowledge Dr MRL Stratford for the analysis of microdialysis samples, Dr SA Hill and Ms TA Griffin for laser Doppler measurements and Dr DH Williamson for invaluable discussions.

### ABBREVIATIONS

$pH_i$, intracellular pH; $pH_e$, extracellular pH; ΔpH, difference between $pH_i$ and $pH_e$; MRS, magnetic resonance spectroscopy; MRI, magnetic resonance imaging; GRE, gradient recalled echo; NTP, nucleoside triphosphate; PME, phosphomonoesters; $P_i$ inorganic phosphate; 3-APP, 3-aminopropyl phosphonate; $lactate_i$, intracellular lactate; $lactate_e$, extracellular lactate.

### REFERENCES

Bergmeyer HU (1974) *Methods of Enzymatic Analysis*, 2nd edn. Verlag Chemie: Weinheim

Brizel DM, Lin S, Johnson JL, Brooks J, Dewhirst MW and Piantadosi CA (1995) The mechanisms by which hyperbaric oxygen and carbogen improve tumour oxygenation. *Br J Cancer* **72**: 1120–1124

Chaplin DJ and Hill SA (1995) Temporal heterogeneity in microregional erythrocyte flux in experimental solid tumours. *Br J Cancer* **71**: 1210–1213

Collingridge DR (1997) Measurement and manipulation of tumour oxygen tension. PhD Thesis, University of London

Dewhirst MW, Ong ET, Rosner GL, Rehmus SW, Shan S, Braun RD, Brizel DM and Secomb TW (1996). Arteriolar oxygenation in tumour and subcutaneous arterioles: effects of inspired air oxygen content. *Br J Cancer* **74**: S241–S246

Dobson GP, Veech RL, Passonneau JV, Kobayashi K, Inubushi T, Wehrli S, Nioka S and Chance B (1992) $^{31}$P NMR and enzymatic analysis of cytosolic phosphocreatine, ATP, $P_i$ and intracellular pH in the isolated working perfused rat heart. *NMR Biomed* **5**: 2–28

Evans RD and Williamson DH (1988) Tissue-specific effects of rapid tumour growth on lipid metabolism in the rat during lactation and on litter removal. *Biochem J* **252**: 65–72

Falk SJ, Ward R and Bleehen NM (1992) The influence of carbogen breathing on tumour tissue oxygenation in man evaluated by computerised $pO_2$ histography. *Br J Cancer* **66**: 919–924

Folbergrova J, MacMillan V and Siesjo BK (1972) The effect of hypercapnic acidosis upon some glycolytic and Krebs cycle-associated intermediates in the rat brain. *J Neurochem* **19**: 2507–2517

Gatenby RA (1995) The potential role of transformation-induced metabolic changes in tumor–host interaction. *Cancer Res* **55**: 4151–4156

Gatenby RA and Gawlinski ET (1996) A reaction–diffusion model of cancer invasion. *Cancer Res* **56**: 5745–5753

Gerweck LE and Seetharaman K (1996) Cellular pH gradient in tumor versus normal tissue: potential exploitation for the treatment of cancer. *Cancer Res* **56**: 1194–1198

Gillies RJ, Liu Z and Bhujwalla Z (1994) $^{31}$P-MRS measurements of extracellular pH of tumors using 3-aminopropylphosphonate. *Am J Physiol* **267**: (Cell Physiol. 36) C195–C203

Grau C, Horsman MR and Overgaard J (1992) Improving the radiation response in a C3H mouse mammary carcinoma by normobaric oxygen or carbogen breathing. *Int J Radiat Oncol Biol Phys* **22**: 415–419

Gullino PM (1976) In vivo utilization of oxygen and glucose by neoplastic tissue. In *Oxygen Transport to Tissue. II.* Grote J, Reneau D and Thaws G (eds), pp. 521–536. Plenum: New York

Haase A, Frahm J, Matthaei D, Hanicke W and Merboldt K-D (1986) FLASH imaging. Rapid NMR imaging using low flip-angle pulses. *J Magn Reson* **67**: 258–266

Hill SA, Collingridge DR, Vojnovic B and Chaplin DJ (1998) Tumour radiosensitisation by high oxygen content gases: influence of the carbon dioxide content of the inspired gas on $pO_2$, microcirculatory function and radiosensitivity. *Int J Radiat Oncol Biol Phys* **40**: 943–951

Hockel M, Schlenger, K, Aral B, Mitze M, Schaffer U and Vaupel P (1996) Association between tumor hypoxia and maligant progression in advanced cancer of the uterine cervix. *Cancer Res* **56**: 4509–4515

Honess DJ and Bleehen NM (1995) Perfusion changes in the RIF-1 tumour and normal tissues after carbogen and nicotinamide, individually and combined. *Br J Cancer* **72**: 1175–1180

Howe FA, Robinson SP and Griffiths JR (1996) Modification of tumour perfusion and oxygenation monitored by gradient recalled echo MRI and $^{31}$P MRS. *NMR Biomed* **9**: 208–216

Kallinowski F, Schlenger KH, Runkel S, Kloes M, Stohrer M, Okunieff P and Vaupel P (1989) Blood flow, metabolism, cellular microenvironment, and growth rate of human tumour xenografts. *Cancer Res* **49**: 3759–3764

Masuda T, Dobson GP and Veech RL (1990). The Gibbs–Donnan near-equilibrium system of heart. *J Biol Chem* **265**: 20321–20334

McCoy CL, Parkins CS, Chaplin DJ, Griffiths JR, Rodrigues LM and Stubbs M (1995) The effect of blood flow modification on intra- and extracellular pH measured by $^{31}$P MRS in murine tumours. *Br J Cancer* **72**: 905–911

McSheehy PJM, Robinson SP, Ojugo ACE, Cannell MB, Leach MO, Judson IR and Griffiths JR (1998) Carbogen breathing increases 5-fluorouracil uptake and cytotoxicity in hypoxic murine RIF-1 tumours: a magnetic resonance study in vivo. *Cancer Res* **58**: 1185–1194

Menke H and Vaupel P (1988) Effect of injectable or inhalational anesthetics and of neuroleptic, neuroleptanalgesic, and sedative agents on tumor blood flow. *Radiat Res* **114**: 64–76

Miller AL, Hawkins RA and Veech RL (1976) Decreased rate of glucose utilisation by rat brain in vivo after exposure to atmospheres containing high concentrations of $CO_2$. *J Neurochem* **25**: 553–558

Nordsmark M, Grau C, Horsman MR, Jorgensen HS and Overgaard J (1995) Relationship between tumour oxygenation, bioenergetic status and radiobiological hypoxia in an experimental model. *Acta Oncol* **34**: 329–334

Nordsmark M, Maxwell RJ, Horsman MR, Bentzen SM and Overgaard J (1997) The effect of hypoxia and hyperoxia on nucleoside triphosphate/inorganic phosphate, $pO_2$ and radiation response in an experimental tumour model. *Br J Cancer* **76**: 1432–1439

Parkins CS, Stratford MRL, Dennis MF, Stubbs M and Chaplin DJ (1997) The relationship between extracellular lactate and tumour pH in a murine tumour model of ischaemia reperfusion. *Br J Cancer* **75**: 319–323

Powell MEB, Hill SA, Saunders MI, Hoskin PJ and Chaplin DJ (1996) Effect of carbogen breathing on tumour microregional blood flow in humans. *Radiother Oncol* **41**: 225–231

Robinson SP, Howe FA and Griffiths JR (1995) Noninvasive monitoring of carbogen-induced changes in tumor blood flow and oxygenation by functional magnetic resonance imaging. *Int J Radiat Oncol Biol Phys* **33**: 855–859

Robinson SP, Rodrigues LM, Ojugo ASE, McSheehy PMJ, Howe FA and Griffiths JR (1997) The response to carbogen breathing in experimental tumour models monitored by gradient-recalled echo magnetic resonance imaging. *Br J Cancer* **75**: 1000–1006

Rodrigues LM, Maxwell RJ, McSheehy PJM, Pinkerton CR, Robinson SP, Stubbs M and Griffiths JR (1997) *In vivo* detection of ifosfamide by $^{31}$P MRS in rat tumours: increased uptake and cytotoxicity induced by carbogen breathing in GH3 prolactinomas. *Br J Cancer* **75**: 62–68

Rojas A (1991) Radiosensitisation with normobaric oxygen and carbogen. *Radiother Oncol* **20** (suppl. 1): 65–70

© Cancer Research Campaign 1998

Sansom JM and Wood PJ (1994) $^{31}$P MRS of tumour metabolism in anaesthetized vs conscious mice. *NMR Biomed* **7**: 167–171

Schwickert G, Walenta S, Sundfor K, Rofstad EK and Mueller-Kleiser W (1995) Correlation of high lactate levels in human cervical cancer with incidence of metastasis. *Cancer Res* **55**: 4757–4759

Song CW, Lee I, Hasegawa T, Rhee JG and Levitt SH (1987) Increase in $pO_2$ and radiosensitivity of tumors by Fluosol-DA (20%) and carbogen. *Cancer Res* **47**: 442–446

Spencer TL and Lehninger A (1976) L-Lactate transport in Ehrlich ascites tumour cells. *Biochem J* **154**: 405–414

Stubbs M, Rodrigues L, Howe FA, Wang J, Jeong KS, Veech RL and Griffiths JR (1994) Metabolic consequences of a reversed pH gradient in rat tumours. *Cancer Res* **54**: 4011–4016

Stubbs M, Veech RL and Griffiths JR (1995) Tumor metabolism: the lessons of magnetic resonance spectroscopy. *Adv Enz Reg* **35**: 101–115

van der Veen JWC, de Beer R, Luyten PR and van Ormondt D (1988) Accurate quantification of *in vivo* $^{31}$P NMR signals using the variable projection method and prior knowledge. *Magn Reson Med* **6**: 92–98

Vaupel P, Schaefer C and Okunieff P (1994) Intracellular acidosis in murine fibrosarcomas coincides with ATP depletion, hypoxia, and high levels of lactate and total Pi. *NMR Biomed* **7**: 128–136

Veech RL (1991) The metabolism of lactate. *NMR Biomed* **4**: 53–58

Warburg O (1930) The metabolism of tumors. Constable: London

Weber G (1968) Carbohydate metabolism in cancer cells and the molecular correlation concept. *Naturwissenshaften* **55**: 418–429

Weber G, Stubbs M and Morris HP (1971) Metabolism of hepatomas of different growth rates in situ and during ischaemia. *Cancer Res* **31**: 2177–2183

© Cancer Research Campaign 1998